# Prognostic value of adipose tissue and muscle mass in advanced colorectal cancer: a post hoc analysis of two non-randomized phase II trials

**DOI:** 10.1186/s12885-019-5319-8

**Published:** 2019-02-12

**Authors:** Nicolas Charette, Caroline Vandeputte, Lieveke Ameye, Camille Van Bogaert, Jonathan Krygier, Thomas Guiot, Amélie Deleporte, Thierry Delaunoit, Karen Geboes, Jean-Luc Van Laethem, Marc Peeters, Gauthier Demolin, Stéphane Holbrechts, Patrick Flamen, Marianne Paesmans, Alain Hendlisz

**Affiliations:** 1 Gastroenterology Department, Hôpital Civil Marie Curie, Charleroi, Belgium; 2 Gastro-Oncology translational laboratory, Institut Jules Bordet - Université Libre de Bruxelles (ULB), Brussels, Belgium; 3 Data centre, Institut Jules Bordet, Université Libre de Bruxelles (ULB), Brussels, Belgium; 4 Medical Oncology Department, Institut Jules Bordet, Université Libre de Bruxelles (ULB), Brussels, Belgium; 5 Nuclear Medicine Department, Institut Jules Bordet, Université Libre de Bruxelles (ULB), Brussels, Belgium; 6 Oncology department, Hôpital de Jolimont, La Louvière, Belgium; 7 Service of digestive oncology, Universitair Ziekenhuis Gent, Ghent, Belgium; 8 Gastroenterology Medico-Surgical Department, Erasme University Hospital, Université Libre de Bruxelles (ULB), Brussels, Belgium; 9 Oncology department, Universitair Ziekenhuis Antwerpen, Antwerpen, Belgium; 10 Gastroenterology Department, Centre Hospitalier Chrétien St-Joseph, Liège, Belgium; 11 Oncology Department, CHU Ambroise Paré, Mons, Belgium; 12 Nuclear Medicine Department, Institut Jules Bordet, Université Libre de Bruxelles (ULB), Brussels, Belgium; 13 Data centre, Institut Jules Bordet - Université Libre de Bruxelles (ULB), Brussels, Belgium; 14 Department of Gastroenterology, CHU de Charleroi, Hôpital Civil Marie Curie, Chaussée de Bruxelles, 140, 6042 Lodelinsart, Belgium

**Keywords:** Colorectal cancer, Prognosis, Chemotherapy, Sarcopenia, Myosteatosis, Obesity, Adipose tissue

## Abstract

**Background:**

The prognostic value of body composition in cancer patients has been widely studied during the last decade. The main finding of these studies is that sarcopenia, or skeletal muscle depletion, assessed by CT imaging correlates with a reduced overall survival (OS). By contrast, the prognostic value of fat mass remains ill-defined. This study aims to analyze the influence of body composition including both muscle mass and adipose tissue on OS in a homogeneous population of advanced colorectal cancer (CRC) patients.

**Methods:**

Among 235 patients with chemorefractory advanced CRC included in the SoMore and RegARd-C trials, body composition was assessed in 217 patients on baseline CT images. The relationship between body composition (sarcopenia, muscle density, subcutaneous and visceral fat index and density), body mass index (BMI) and OS were evaluated.

**Results:**

Patients with a higher BMI had a better OS (≥30 versus < 30, HR: 0.50; 0.33–0.76). Those with low muscle index and muscle density had an increased mortality (HR: 2.06; 1.45–2.93 and HR: 1.54; 1.09–2.18, respectively). Likewise, low subcutaneous and visceral fat index were associated with an increased risk of dying (HR: 1.63; 1.23–2.17 and 1.48; 1.09–2.02 respectively), as were a high subcutaneous and visceral adipose tissue density (HR: 1.93; 1.44–2.57 and 2.40; 1.79–3.20 respectively). In multivariate analysis, a high visceral fat density was the main predictor of poor survival.

**Conclusions:**

Our results confirm the protective role of obesity in CRC patients at an advanced stage, as well as the negative prognostic impact of muscle depletion on survival. More importantly, our data show for the first time that visceral adipose tissue density is an important prognostic factor in metastatic CRC.

**Trial registration:**

NCT01290926, 07/02/2011 and NCT01929616, 28/08/2013.

## Background

Malnutrition has been known for more than 30 years to be associated with reduced overall survival (OS) of cancer patients [1] and is present in one third of colorectal cancer (CRC) cases [2]. On the other hand, the prevalence of overweight and obesity has dramatically increased during the last 3 decades [3] and obesity is now widely recognized as a risk factor for several cancer types, including CRC [4]. As a result, the clinical picture of malnutrition in cancer patients has changed and recent diagnostic criteria for cancer cachexia emphasize on loss of muscle mass, known as sarcopenia, along with weight loss and low body mass index (BMI) [5]. Accordingly, several techniques have been developed in order to evaluate body composition. Among these, dual X-ray absorptiometry (DEXA) is considered the gold standard but is rarely used in clinical practice where bioelectrical impedance analysis and computed tomography (CT)-based regional body composition analysis are more readily available [6].

CT-based regional analysis of muscle and adipose tissue at the level of the third lumbar vertebra strongly correlates with whole-body fat and muscle mass [7], making it an attractive way to evaluate body composition in cancer patients, since anyhow CTs are routinely performed during their follow up. Moreover, the mean muscle attenuation expressed in Hounsfield units (HU) also gives a qualitative information, a lower attenuation being associated with a higher muscle lipid content [8]. Skeletal muscle depletion as assessed by CT imaging has been associated with a reduced OS in multiple studies [9]. By contrast, little is known regarding the prognostic impact of adipose tissue area and density in cancer patients, even though these parameters are easily measured on the CT images used for the evaluation of skeletal muscle [7].

The aim of this study was to assess the association of BMI, skeletal muscle and adipose tissue mass and density with OS in a homogenous group of patients with chemorefractory advanced CRC.

## Methods

### Patient population

All patients included in the SoMore (NCT01290926, 07/02/2011) [10] and the RegARd-C (NCT01929616, 28/08/2013) [11] clinical trials were evaluated in the present study. Both studies were single arm, prospective, open label, non-randomized, multicenter clinical trials, assessing the prognostic significance of early metabolic response to treatment in chemorefractory metastatic CRC patients. The SoMore study enrolled 97 patients between February and October 2011 to receive capecitabine in combination with sorafenib. For RegARd-C, 138 patients were enrolled between August 2013 and August 2014 and received regorafenib as a monotherapy. Eligible patients for both trials had progressive disease after treatment with 5FU, oxaliplatin, irinotecan as well as bevacizumab and anti-EGFR antibodies as appropriate. Patients with an ECOG performance status > 1, i.e. patients unable to carry out light or sedentary work, were not eligible. All patients received a baseline ^18^F-fluorodeoxyglucose (FDG)-positron emission tomography (PET) combined with CT. After exclusion of patients without follow up data allowing determination of OS or whose CT was not appropriate for body composition evaluation, 88 patients (91%) of the SoMore study and 129 patients (93%) of the RegARd-C study were included in the final analysis. Overall survival in months was defined as the time from inclusion to the date of death from any cause. Given the similar inclusion criteria and the fact that regorafenib and sorafenib are closely related multikinase inhibitors with limited benefit in our study population [10, 12], the 217 eligible patients were treated as a single patient population.

### Body mass index and CT-based body composition analysis

**Fig. 1:**
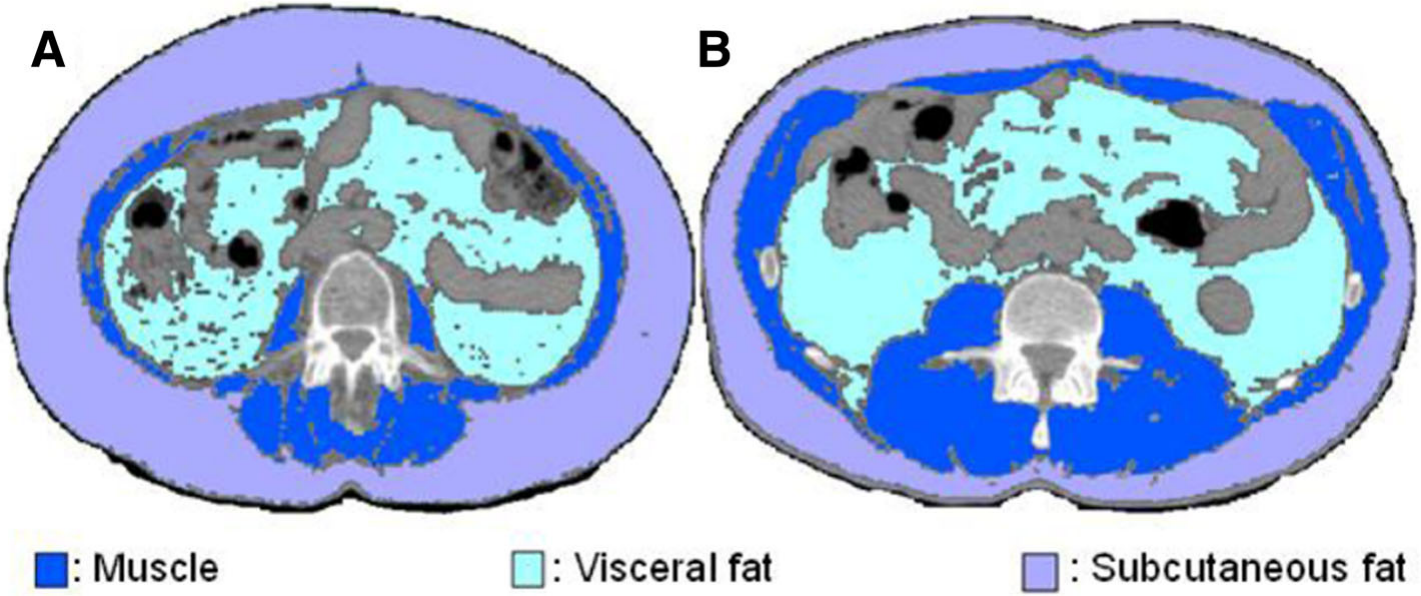
CT images of the third lumbar vertebra region in a sarcopenic (a) and a non-sarcopenic (b) patient

BMI (kg/m^2^) was calculated as weight in kilograms divided by the square of height in meters. BMI was then categorized according to the World Health Organization modified by Martin et al. (underweight < 20.0, normal weight 20.0–24.9, overweight 25.0–29.9, obesity ≥30.0) [13]. Evaluation of body composition was performed using the PLANET Onco® software (DOSIsoft, Cachan, France) by two independent investigators. The cross-sectional area (cm^2^) of skeletal muscle (psoas, paraspinal and abdominal wall muscles), subcutaneous adipose tissue and visceral adipose tissue was delineated on two adjacent CT slices at the level of the third lumbar vertebra. Skeletal muscle, subcutaneous adipose tissue and visceral adipose tissue were defined by ranges of − 29 to 150, − 190 to − 30 and − 150 to − 50 HU, respectively [7]. Muscle and fat areas (cm^2^) were normalized for the square of height (m^2^) as indexes (cm^2^/m^2^). Figure 1 shows an example of the resulting images in a sarcopenic and a non-sarcopenic patient. Additionally, the mean attenuation (HU) for each area was recorded. Interobserver agreement was excellent for all body composition parameters with *r*^2^ ranging from 0.95 to 1.0.

Sarcopenia was defined as a skeletal muscle index < 41 in women, < 43 in men with a BMI < 25, and < 53 in men with a BMI ≥ 25. These cut offs have been validated in a large population of cancer patients [13]. Since there is no validated definition of low fat mass in cancer patients based on CT-imaging, an optimal cutoff was determined in our study population.

### Statistical analysis

The Mann-Whitney-Wilcoxon test and the chi-square or Fisher exact test were used to assess differences in continuous and categorical variables, respectively. The association between two continuous variables was assessed with Spearman correlation. To assess the heterogeneity between SoMore or RegARd-C, we used the chi-square test.

The primary outcome was OS. To assess whether gender-specific cutoffs or BMI-specific cutoffs for each of the six body composition variables were needed, we fitted a multivariate Cox’s proportional hazards model containing, that variable, gender, BMI (≤25 vs > 25) and the two two-way interactions (variable*gender, variable*BMI25) and the three-way interaction (variable*gender*BMI25). We performed backward variable selection. If e.g. the interaction variable*gender would be retained, this would indicate that the effect of the variable on OS depends on the gender, and thus stratifying by gender would be of interest when calculating the optimal cutoff.

The optimal cutoff in each of the six body composition parameters was determined by the SASmacro %*findcut* [14]. The outer 20% of the continuous variable distribution were excluded in this analysis to avoid having small numbers in one of the groups following dichotomization, to prevent substantial losses in statistical power.

For the univariate analysis, Kaplan-Meier curves were used to compare OS of patients below or above the optimal cutoff determined for each body composition parameter. The hazard ratio (HR) and 95% confidence interval (CI) were calculated using Cox’s proportional hazards model, and logrank tests were used to compare survival curves.

For the multivariate analysis, a stepwise variable selection was performed, considering the study subset (SoMore vs RegARd-c), age, BMI (4 categories), gender, performance status, time interval between diagnosis and inclusion in the respective study (SoMore or RegARd-c), low skeletal muscle index, low muscle density, low subcutaneous adipose tissue index, high subcutaneous adipose tissue density, low visceral adipose tissue index and high visceral adipose tissue density. Results were considered statistically significant at the bilateral *p* < 0.05 level. SAS version 9.4 was used for all statistical analyses.

This study has been approved by the ethics committee of Institut Jules Bordet and has been performed in accordance with the ethical standards laid down in the 1964 Declaration of Helsinki and its later amendments.

## Results

### Patient characteristics

Among the 217 subjects included in the present study, 94 (43%) were women and 123 (57%) were men. Median BMI was 24.9 (14.1–41.0) with 49% of these heavily pre-treated patients being overweight or obese. Sarcopenia was present in 150 (69%) of the study population. Although sarcopenia was less prevalent in obese patients as compared to non-obese, it was still observed in 48% of this subgroup. Table 1 shows the baseline characteristics of the patients in both study subsets.

**Table 1:** Patients characteristics

	SoMore (*N* = 88)		RegARd-C (*N* = 129)		Total (*N* = 217)	
Age						
Mean ± std	61 ± 10		65 ± 11		63 ± 11	
Median (min-max)	63 (28 to 83)		67 (32 to 85)		65 (28 to 85)	
Gender						
Female	38	43%	56	43%	94	43%
Male	50	57%	73	57%	123	57%
BMI						
Mean ± std	25.3 ± 4.7		25.6 ± 5.0		25.5 ± 4.9	
Median (min-max)	25.4 (16.5 to 35.6)		24.4 (14.1 to 41.0)		24.9 (14.1 to 41.0)	
ECOG PS						
0	49	56%	63	49%	112	52%
1	39	44%	66	51%	105	48%
Years between diagnosis and inclusion in the trial						
Mean ± std	3.3 ± 2.6		3.7 ± 2.6		3.5 ± 2.6	
Median (min-max)	2.3 (0.2 to 14.9)		3.0 (0.1 to 13.0)		2.6 (0.1 to 14.9)	

At the time of analysis, 197 (90%) of the patients had died. Median OS (mOS) was 8.2 months (6.8–10.4) in the SoMore study and 7.1 months (3.4 to 13.3) in the RegARd-C study. To make sure that data from these 2 trials could be pooled, a test for heterogeneity comparing SoMore and RegARd-C patients was performed for each body composition variable dichotomized considering the gender-specific median in each dataset (Table 2). This test found no evidence that the effect of body composition on OS could be dependent of the considered dataset, allowing us to pool the data for further evaluation.

**Table 2:** Test for heterogeneity comparing SoMore and RegARd-C

	SoMore	RegARd-c	*P*-value Test heterogeneity
	Hazard ratio (95% CI)	Hazard ratio (95% CI)	
Muscle index (low vs high)	1.55 (1.00 to 2.38)	1.86 (1.28 to 2.72)	0.53
Muscle density (low vs high)	0.74 (0.48 to 1.14)	1.12 (0.78 to 1.63)	0.15
Subcutaneaous fat index (low vs high)	1.08 (0.70 to 1.66)	1.81 (1.24 to 2.64)	0.08
Subcutaneous fat density (low vs high)	0.64 (0.41 to 0.98)	0.53 (0.37 to 0.77)	0.52
Visceral fat index (low vs high)	1.21 (0.79 to 1.86)	1.59 (1.09 to 2.31)	0.35
Visceral fat density (low vs high)	0.60 (0.39 to 0.92)	0.42 (0.28 to 0.61)	0.23

Hazard ratio for OS were calculated for muscle index, muscle density, subcutaneous fat index, subcutaneous fat density, visceral fat index and visceral fat density comparing patients above and below the gender-specific median in each dataset. A test for heterogeneity comparing the two datasets for each body composition parameter was then performed and did not reach statistical significance

A wide variation in body composition was found between men and women. Women had a lower BMI (mean ± SD, women: 24.5 ± 5.5 vs men: 26.3 ± 4.1, *p* < 0.001) and skeletal muscle index (women: 36.2 ± 5.8 vs men: 46.6 ± 9.2, *p* < 0.001). Men had a lower subcutaneous fat index (women: 74.8 ± 45.2 vs men: 56.2 ± 29.9, *p* = 0.002) but a higher visceral fat index (women: 29.8 ± 26.2 vs men: 55.9 ± 33.7, *p* < 0.001) and lower visceral adipose tissue density (women: − 87.4 ± 10.1 vs men: − 90.8 ± 8.9, *p* = 0.006). BMI was significantly correlated with skeletal muscle index (*r* = 0.49; *p* < 0.001), subcutaneous fat index (*r* = 0.73; *p* < 0.001), and visceral fat index (*r* = 0.75; *p* < 0.001) but was inversely correlated with muscle density (*r* = − 0.36; *p* < 0.001), subcutaneous fat density (*r* = − 0.60; *p* < 0.001), and visceral fat density (*r* = − 0.62; *p* < 0.001).

### Survival and optimal stratification

In univariate analysis, obesity was associated with an increase in OS (BMI ≥30 vs < 30, HR for mortality 0.50 (0.33–0.76)). Neither the presence of sarcopenia nor a low muscle density according to a validated cutoff [13] were associated with survival (HR 1.28 (0.93–1.75) and 1.25 (0.89–1.77), respectively).

However, a skeletal muscle index below the gender-specific median was associated with increased mortality in both subsets, as shown in Table 2, suggesting that muscle loss had a prognostic impact indeed. Since published cutoffs for skeletal muscle index and muscle density failed to predict mortality in our cohort and no validated cutoffs for subcutaneous fat index, subcutaneous fat density, visceral fat index or visceral fat density could be found in the existing literature, the optimal cutoff associated with lower survival was assessed for each body composition variable. Gender and BMI were taken into account in the model but no statistically significant interaction between these variables and body composition were found, thus alleviating the need to stratify the population by gender or BMI. The optimal cutoff for association with OS for each body composition variable is shown in Table 3.

**Table 3:** Optimal cutoff for association with OS

	Optimal cutoff for association with OS	high N (%)	low N (%)
Skeletal muscle index	47.5	54 (25%)	163 (75%)
Muscle density	22.5	175 (81%)	42 (19%)
Subcutaneous fat index	50	129 (59%)	88 (41%)
Subcutaneous fat density	-100	110 (51%)	107 (49%)
Visceral fat index	60	64 (29%)	153 (71%)
Visceral fat density	−90	102 (47%)	115 (53%)

Skeletal muscle index, subcutaneous fat index, visceral fat index are expressed in cm^2^/m^2^; muscle density, subcutaneous fat density, visceral fat density are expressed in HU

**Table 4:** Multivariate analysis of survival

		Hazard ratio (95% CI)	*P*-value
BMI	obese	0.60 (0.38 to 0.95)	0.03
Years diagnosis till inclusion	Per one-year	0.89 (0.84 to 0.95)	< 0.001
Skeletal muscle index	Low, < 47.5	1.49 (1.04 to 2.15)	0.03
Muscle density	Low, < 22.5	1.80 (1.24 to 2.61)	0.002
Visceral fat density	High, ≥90	1.87 (1.38 to 2.54)	< 0.001

Obese: BMI ≥ 30 kg/m^2^; skeletal muscle index expressed in cm^2^/m^2^; muscle and visceral fat density expressed in HU

**Fig. 2:**
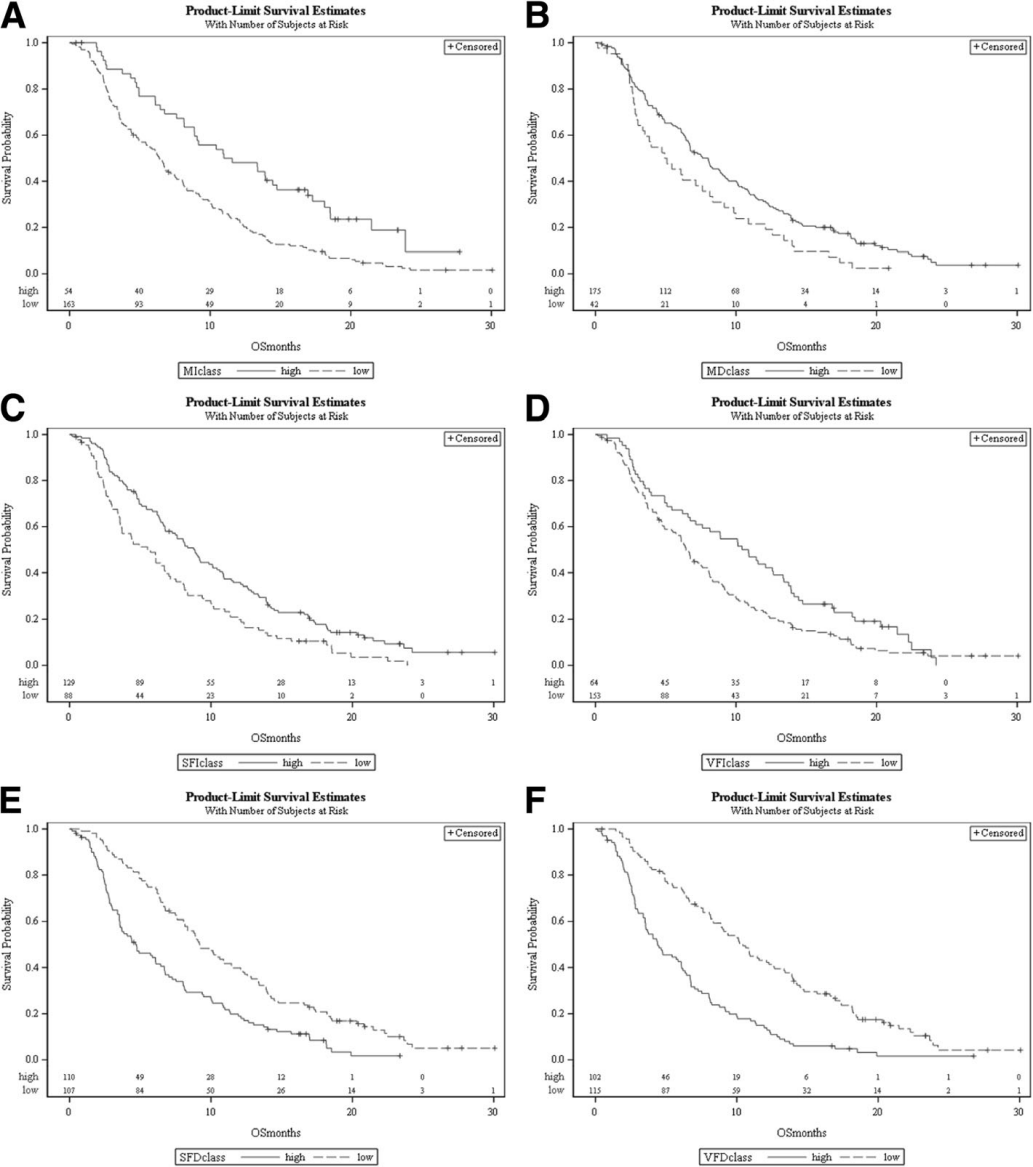
Survival curves based on skeletal muscle index (**a**), muscle density (**b**), subcutaneous fat index (**c**), visceral fat index (**d**), subcutaneous fat density (**e**) and visceral fat density (**f**). Patients with skeletal muscle index and muscle density below the thresholds had an increased mortality. Low subcutaneous and visceral fat index were also associated with an increased risk of dying. Finally, a high subcutaneous and visceral fat density also correlated with mortality

Patients with a skeletal muscle index and muscle density below these thresholds had an increased mortality, with HR of 2.06 (1.45–2.93) and 1.54 (1.09–2.18), respectively. Low subcutaneous and visceral fat index were also associated with an increased risk of dying, with hazard ratios of 1.63 (1.23–2.17) and 1.48 (1.09–2.02), respectively. Finally, a high subcutaneous and visceral fat density were also correlated with mortality, with a HR of 1.93 (1.44–2.57) and 2.40 (1.79–3.20), respectively. The corresponding survival curves are shown in Fig. 2 A-F.

### Multivariate analysis

A multivariate analysis was then performed, taking into account the population subset (SoMore vs RegARd-C study participants), age, BMI (obese, overweight, normal, underweight), gender, performance status, time interval between diagnosis and inclusion in the study (SoMore or RegARd-C), low skeletal muscle index, low muscle density, low subcutaneous fat index, high subcutaneous fat density, low visceral fat index, high visceral fat density. Results are shown in Table 4 and demonstrate that BMI (obese or not), time interval from diagnosis till inclusion, low skeletal muscle index, low muscle density and high visceral fat density are independently and significantly associated with a reduced OS. Moreover, the main factor predicting survival seems to be high visceral fat density.

## Discussion

In this study, we measured skeletal muscle mass as well as subcutaneous and visceral fat mass in a population that is highly homogeneous from an oncologic perspective. Indeed, all patients had been heavily pretreated for a metastatic CRC and received similar although not identical treatments in both study cohorts. More than two thirds of these selected patients were sarcopenic, which is in line with the results of another study assessing the presence of sarcopenia in metastatic CRC patients [15] but much higher than the 39% of sarcopenic patients found in a cohort of early stage CRC patients surgically treated with curative intent [16]. Our results also dramatically differ from the 31.2% prevalence of malnutrition found in CRC patients using weight loss and BMI but not body composition [2], highlighting the need to integrate body composition parameters in the nutritional evaluation of these patients. However, sarcopenia as defined by validated cutoffs was not correlated with OS. This is unlikely to be due to a lack of statistical power considering our sample size and number of events. Alternatively, one may hypothesize that the use of different software could lead to slightly different results. This is also unlikely because a recent study found an excellent agreement for the diagnosis of sarcopenia based on several softwares [17]. Since the cutoffs for sarcopenia have been derived from a large population comprising patients with different cancer types at various stages of their disease [13], it may be hypothesized that these cutoffs do not fit our highly homogeneous population of very advanced colorectal cancer patients. Conversely, our cutoffs for sarcopenia may thus not be generalized to other patient populations. Nonetheless, our results clearly show that a reduced muscle mass correlates with a reduced survival in CRC patients which is in line with results found in mixed cancer patients population [9, 13].

Another important finding of our study is the association of obesity with an increased OS, which is a typical example of the obesity paradox. Indeed, obesity is a known risk factor for colon cancer incidence and mortality in healthy subjects [4, 18]. The relation between survival and the presence of obesity after a diagnosis of CRC is less clear. In stage II and III CRC, obese patients have a reduced OS [19]. By contrast, obesity was not associated with a reduced OS in patients with metastatic CRC undergoing chemotherapy [20]. The obesity paradox is often considered to reflect statistical bias and reverse causality rather than an established biological phenomenon [21]. The *post-hoc* nature of our analysis does not allow us to exclude such bias. However, our data were prospectively collected and obesity was found to predict OS in a multivariate analysis taking several other body composition-related factors into account. Moreover, several prognostic models in mixed cancer patient populations show a protective role of overweight and/or obesity [13, 22]. Another potential explanation to the obesity paradox in cancer patients is the failure of most of the studies exploring the relation of BMI and survival to take the body composition into account. The general finding when muscle mass is considered is that obesity in not associated with a better OS in the presence of low muscle mass. The low prevalence of sarcopenia in obese patients could thus account for the better prognosis associated with high BMI [23]. Indeed, several studies have found a low prevalence of sarcopenia in obese patients. One study evaluating 995 patients at hospital admission found sarcopenia in only 1% of obese patients [24]. In another study evaluating obese patients with colorectal or lung cancer, the prevalence of sarcopenia was only 15% [25]. Similarly, another study in obese CRC patients undergoing surgery found sarcopenia in 16% of the cases [16]. Most of the patients in these two studies had no metastases. By contrast, sarcopenia was present in 48% of obese patients in our cohort, and obesity was still associated with a better survival in a multivariate model taking muscle mass into account, making this last explanation unlikely in our study. Therefore, we think that our observation regarding obesity and survival is not a statistical artefact. One explanation could be a different role of obesity depending on the stage of the disease where the adverse metabolic and inflammatory status takes precedence in early disease stages whereas the larger amount of energy stored in adipose tissue becomes increasingly important in advanced disease.

While the prognostic impact of low muscle mass has been shown in numerous studies, the role of adipose tissue mass and density has received much less interest, and research on this topic has yielded conflicting results. For instance, a high visceral fat area was associated with a shorter disease free survival in breast cancer patients treated with neoadjuvant chemotherapy [26]. By contrast, patients with a high visceral fat area and high visceral fat density had a longer time to biochemical recurrence after curative treatment of prostate cancer, although their predictive value was lost in a multivariate model including all risk factors grouped together according to the CAPRA-S score [27]. Similarly, both low and high visceral adipose tissue index have been associated with a reduced OS in patients receiving immunochemotherapy for diffuse large B-cell lymphoma [28, 29]. In CRC, a high visceral fat area has been associated with a shorter OS in patients treated with bevacizumab but not in those treated with chemotherapy alone [30]. In our study, both a low visceral and subcutaneous fat index were associated with a reduced OS only in univariate analysis but failed to reach statistical significance in multivariate analysis. However, our results point to an important prognostic role of the adipose tissue. Indeed, high visceral fat density was strongly associated with a reduced survival. To the best of our knowledge, no previous study has assessed the prognostic role of fat density in metastatic cancer patients. However, higher adipose tissue density was associated with mortality in two large cohorts of healthy older adults [31]. As for muscle density, adipose tissue density might be a qualitative marker whereas adipose tissue index reflects the quantity of adipose tissue. Theoretically, a higher fat density could reflect a depletion of fat storage associated with poor nutritional status. Indeed, in one study in cancer patients and rats, the radiologic density of brown adipose tissue increased with activation. In rats, this higher brown adipose tissue density was associated histologically with a lower lipid content [32]. Higher adipose tissue density has also been associated with smaller adipocytes in non-human primates [31]. Alternatively, the subcutaneous adipose tissue of cachectic patients with gastro-intestinal cancer is characterized by fibrosis and inflammatory cell infiltration [33]. Although no radiological correlations were made in this study, fibrosis and inflammation should also lead to increased adipose tissue density. Another explanation for a higher adipose tissue density could be a browning of the adipose tissue, a phenomenon involved in cancer cachexia [34]. Interestingly, a cutoff of − 88 HU has been proposed to differentiate brown from white adipose tissue [35], which is close to our optimal cutoff for association with OS of − 90 HU. Whether the prognostic impact of adipose tissue density in our study is mediated by malnutrition, browning of the adipose tissue, the role of inflammation and/or altered adipokines remains to be investigated.

## Conclusion

Our results confirm the protective role of obesity in cancer patients at an advanced stage, as well as the negative prognostic impact of low muscle index and muscle density. Finally, and more importantly, this work shows for the first time in metastatic cancer patients that visceral adipose tissue density is an important prognostic factor even when well-known oncologic prognostic variables such as performance status and length of disease are taken into account. Further research is needed to confirm these findings regarding adipose tissue, which may help to better define the prognosis of advanced colorectal cancer patients in the future.

## Abbreviations

BMI: Body mass index
CRC: colorectal cancer
HU: Hounsfield units
OS: Overall survival
